# Epidemiology and antifungal susceptibility of candidemia isolates of non-*albicans Candida* species from cancer patients

**DOI:** 10.1038/emi.2017.74

**Published:** 2017-10-11

**Authors:** Ping-Feng Wu, Wei-Lun Liu, Min-Han Hsieh, Ing-Moi Hii, Yu-Lin Lee, Yi-Tsung Lin, Mao-Wang Ho, Chun-Eng Liu, Yen-Hsu Chen, Fu-Der Wang

**Affiliations:** 1 Division of Infectious Diseases, Department of Medicine, Taipei Veterans General Hospital, Taipei 112, Taiwan; 2 School of Medicine, National Yang-Ming University, Taipei 112, Taiwan; 3 Department of Intensive Care Medicine, Chi Mei Medical Center, Liouying, Tainan 736, Taiwan; 4 College of Medicine, Fu Jen Catholic University, New Taipei City 242, Taiwan; 5 Division of Infectious Diseases, Department of Internal Medicine, Kaohsiung Medical University Hospital, Kaohsiung Medical University, Kaohsiung 807, Taiwan; 6 Section of Infectious Diseases, Department of Internal Medicine, Changhua Christian Hospital, Changhua 500, Taiwan; 7 Institute of Emergency and Critical Care Medicine, National Yang-Ming University, Taipei 112, Taiwan; 8 Division of Infectious Diseases, Department of Internal Medicine, China Medical University Hospital, China Medical University, Taichung 404, Taiwan; 9 Department of Biological Science and Technology, College of Biological Science and Technology, National Chiao Tung University, HsinChu 300, Taiwan

**Keywords:** cancer, epidemiology, non-*albicans* candidemia, susceptibility

## Abstract

Candidemia is a growing concern worldwide, and its species distribution has shifted toward non-*albicans Candida* in recent decades, especially in patients with malignancy. This study aimed to update the epidemiology and antifungal susceptibility of non-*albicans* candidemia isolates from the cancer patients. Adult cancer patients with non-*albicans* candidemia were recruited, and clinical data were retrospectively collected from five medical centers in Taiwan from 1 July 2011 to 30 June 2014. *In vitro* susceptibility was determined by the broth dilution method using a Sensititre YeastOne system and interpreted according to the guidelines of the Clinical and Laboratory Standards Institute. A total of 346 episodes of non-*albicans* candidemia were identified in cancer patients. *Candida tropicalis* was the most common species (*n*=145, 41.9%) and had the highest resistance rate to fluconazole (*n*=17, 13.9%) among all the preserved isolates, including *C. tropicalis*, *Candida glabrata* and *Candida parapsilosis*. A higher Charlson comorbidity index, non-*albicans* candidemia due to *C. tropicalis*, neutropenia and septic shock were independent predictors of 28-day mortality. In conclusion, the species distribution and antifungal susceptibility of non-*albicans* candidemia isolates in our study differed from those in Western countries, providing useful information about local epidemiology for the selection of empirical antifungal agents for cancer patients.

## INTRODUCTION

*Candida* species have become increasingly important pathogens in healthcare settings worldwide. In 2004, candidemia was reported as the fourth to sixth leading cause of nosocomial bloodstream infections in Western countries, including in the United States and Switzerland.^1, 2^ A more recent multicenter study in 2014 that enrolled 183 hospitals in the United States showed that candidemia was the most common etiology of healthcare-associated bloodstream infections.^3^ In Taiwan, the incidence of candidemia has also shown an increasing trend, from 0.8 patients per 10 000 discharges in 1981 to 28.8 patients per 10 000 discharges in 2000.^4^ Furthermore, the species distribution of candidemia varies geographically and has changed in recent decades. The proportion of different species causing candidemia has shifted toward non-*albicans Candida* species; *Candida tropicalis* and *Candida parapsilosis* are prominent in Taiwan and China, respectively,^5, 6, 7^ and *Candida glabrata* is prominent in the United States.^8, 9^

Malignancy is a major underlying comorbidity of patients with candidemia. For cancer patients, candidemia is associated with a high mortality rate (30%–50%) that results in substantial healthcare costs and prolonged hospital stays.^10, 11, 12, 13^ Recent literature has shown that despite the introduction of novel antifungal agents, candidemia in cancer patients remains a severe disease with a high mortality rate of 31–68%.^6, 14, 15, 16^ Candidemia caused by fluconazole-resistant strains, which are composed exclusively of non-*albicans Candida* species, has been shown to significantly increase mortality among patients with solid organ or hematological malignancies.^14^ Furthermore, previous studies have reported that the contribution of non-*albicans Candida* species to candidemia in cancer patients is increasing.^16, 17, 18^ Hence, an understanding of the epidemiology of non-*albicans* candidemia and the antifungal susceptibilities of these isolates is currently imperative to guide optimal empirical treatment strategies for affected patients.

In this report, we conducted a multicenter study to determine the clinical characteristics and antifungal susceptibilities of non-*albicans* candidemia in cancer patients and to identify the potential risk factors associated with 28-day mortality.

## MATERIALS AND METHODS

### Setting and study design

This multicenter study was conducted at the Taipei Veterans General Hospital with 2900 beds in northern Taiwan, Chi Mei Hospital with 900 beds in southern Taiwan, Kaohsiung Medical University Hospital with 1600 beds in southern Taiwan, China Medical University Hospital with 2111 beds in central Taiwan and Changhua Christian Hospital with 1487 beds in central Taiwan. All adult cancer patients older than 20 years of age, with a bloodstream infection due to non-*albicans Candida*, and who were admitted to any of the above-mentioned five medical centers between 1 July 2011 and 30 June 2014 were enrolled in the study. In the case of patients who suffered two or more episodes of non-*albicans* candidemia during the study period, only the first episode was included in our analysis. Patients with a bloodstream infection caused by two or more *Candida* species were excluded. Non-*albicans Candida* isolates obtained from patients at the enrolled hospitals were analyzed for antifungal susceptibility at a medical center.

The medical records of all enrolled patients were reviewed by infectious disease specialists, and the variables collected from medical charts were as follows: age, gender, Charlson comorbidity index, underlying comorbidities, cancer types, origin of non-*albicans* candidemia, underlying conditions (use of a central venous catheter, recently received chemotherapy, total parenteral nutrition, steroid, proton-pump inhibitor and abdominal surgery, and prior exposure to broad-spectrum antibiotics), severity of illness (neutropenia and septic shock), concomitant bacterial infections, antifungal therapy and 28-day outcome. This study was approved by the Medical Ethics Committees of the five enrolled hospitals.

### Definitions

Patients infected with non-*albicans* candidemia were confirmed by at least one set of positive blood cultures and compatible clinical symptoms and signs.^19^ The origin of the candidemia was identified as the site from which the same non-*albicans Candida* species was isolated. Catheter-related bloodstream infection (CRBSI) was defined by semi-quantitative tip culture as the growth of ≥15 colonies that were identical to the species identified from the peripheral blood culture.^20^ Primary candidemia was defined as a non-*albicans* candidemia occurring in the absence of an apparent portal of entry. Healthcare-associated infection was determined according to the criteria provided by the Centers for Disease Control and Prevention, USA.^21^ Recent abdominal surgery, the recent use of steroids (at least 10 mg of prednisolone or an equivalent daily dosage for more than 7 days), broad-spectrum antibiotics or proton-pump inhibitors were defined as undergoing interventions or agents when they occurred within 30 days before the positive blood culture. Chemotherapy received within 90 days before the infection was defined as ‘recently received chemotherapy’. Neutropenia was indicated by an absolute neutrophil count of <500 × 10^6^ cells per liter, and septic shock was defined by a systolic pressure below 90 mm Hg and a need for vasopressors. Antifungal therapy was determined based on the predominant agent, which was used for most of the duration of the treatment course. The 28-day mortality values were identified based on the medical records of the five enrolled hospitals, including the discharge day before 28 days from the onset of non-*albicans* candidemia.

### Microbiological analysis of non-*albicans Candida* isolates

All preserved, available non-*albicans Candida* isolates were identified by morphology analysis on CHROMagar (Creative Life Science, New Taipei City, Taiwan) and biochemical methods using a Vitek 2 system with the YST Card (bioMérieux, Durham, NC, USA). The broth microdilution method using a Sensititre YeastOne system (Trek Diagnostic Systems, East Grinstead, UK) was performed to determine the minimum inhibitory concentration (MIC) according to the manufacturer’s instructions. *C. krusei* ATCC 6258 and *C. parapsilosis* ATCC 22019 were used as quality control strains. The MICs of amphotericin B, caspofungin, micafungin, anidulafungin, fluconazole, voriconazole, itraconazole, posaconazole and flucytosine were determined, and interpretation criteria were based on the MIC breakpoints of non-*albicans Candida* species, as recommended by the Clinical and Laboratory Standards Institute.^22^

### Statistical analyses

Statistical analyses were performed using SPSS software version 17 (SPSS, Chicago, IL, USA). Categorical data were compared using *χ*^2^ or Fisher’s exact tests. Continuous data were analyzed using Student *t*-test or Mann–Whitney *U* test. Statistical significances were determined using two-tailed tests, and a value of *P*<0.05 was considered significant. Logistic regression analysis was used to identify the independent predictors of 28-day mortality. All biologically plausible variables with values of *P*<0.10 in univariate testing were included in the model.

## RESULTS

**Figure 1:**
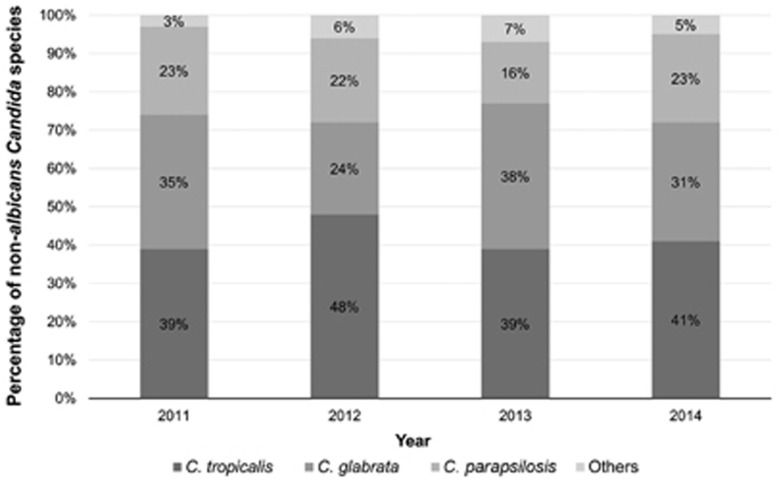
Trend in species distribution of non-*albicans* candidemia from 2011 to 2014.

During the study period, 346 episodes of candidemia caused by non-*albicans Candida* were identified in a study population of 346 patients. No significant differences were observed in the proportions of candidemia caused by different non-*albicans Candida* species each year (*P*=0.489). In addition, Figure 1 shows the species ratio trends of non-*albicans* candidemia cases from 2011 to 2014. Table 1 summarizes the demographics and clinical data of the patients with non-*albicans* candidemia enrolled in the study. The study population included 221 (63.9%) male and 125 (36.1%) female participants, and the mean age of all patients enrolled was 63.9±15.4 years old. Gastrointestinal cancer was the most common malignancy in patients with non-*albicans* candidemia enrolled in the study (160 patients, 46.2%). Apart from malignancy, 168 (48.6%) patients had other underlying comorbidities; diabetes mellitus (107 patients, 30.9%) was the most frequent disease among these conditions. CRBSI (108 patients, 31.2%) accounted for the most commonly identified origin of non-*albicans* candidemia, and 136 (39.3%) episodes were classified as primary infection. The presence of a central venous catheter (294 patients, 85%) and prior exposure to broad-spectrum antibiotics (306 patients, 88.4%) were the most common risk factors associated with non-*albicans* candidemia in this study. The majority of predominant antifungal agents were triazoles (192 patients, 55.5%). Across the study population, only 29 (8.4%) and 83 (24%) patients ever received prophylactic or empirical antifungal therapies, respectively. In addition to the patients undergoing prophylactic or no treatment, the median (interquartile range) duration of prescribed antifungal agents after collecting index blood cultures was two (0.20–3.18) days.

Table 2 shows the distribution and frequency of non-*albicans Candida* species isolated from patients with different types of cancers. *C. tropicalis* was the major cause of non-*albicans* candidemia in all patients, regardless of cancer type. However, no significant differences were observed in the distribution of non-*albicans Candida* species among the five major cancer categories analyzed. Notably, when patients with solid organ malignancies were compared with those with hematological cancers, non-*albicans* candidemia caused by *C. tropicalis* was significantly higher in patients with hematological malignancies (58.7% versus 39.3%, *P*=0.020).

A total of 251 non-*albicans* candidemia isolates were available for the antifungal susceptibility test, as shown in Table 3. Of these non-*albicans* candidemia-causative isolates, 22 (8.8%) were resistant to *in vitro* fluconazole, including 17 *C. tropicalis*, 3 *C. glabrata* and 2 *C. parapsilosis* isolates. Although *C. tropicalis* showed the highest rate of resistance to fluconazole (13.9%) among the preserved isolates, *C. glabrata* exhibited the highest fluconazole MIC_50_ and MIC_90_ (16 mg/L and 32 mg/L, respectively) among all non-*albicans Candida* species. The overall susceptibility rates for echinocandins were high (>90%), except for caspofungin in *C. glabrata* (83.3%) isolates. The echinocandin MIC_50_ and MIC_90_ of *C. parapsilosis* (0.5–1 mg/L and 0.5–2 mg/L) were higher than other non-*albicans Candida* species, but all *C. parapsilosis* isolates were susceptible to three echinocandins.

The overall 28-day mortality rate in this study was 44.5% (154 patients). Moreover, the mortality rates among the three most common non-*albicans Candida* species, *C. tropicalis*, *C. glabrata* and *C. parapsilosis*, were 53.8% (78 out of 145 patients), 40.5% (45 out of 111 patients) and 31.4% (22 out of 70 patients), respectively. To identify the independent risk factors associated with 28-day mortality in cancer patients with non-*albicans* candidemia, we stratified the 346 patients into survival and death groups (Table 4). In the univariate analysis, patients with a higher Charlson comorbidity index (*P*=0.011), a non-*albicans* candidemia caused by *C. tropicalis* (*P*=0.003), a primary infection (*P*=0.006), neutropenia (*P*=0.005) or septic shock (*P*<0.001) had significantly higher 28-day mortality rates. The results of this multivariate analysis showed that a higher Charlson comorbidity index (odds ratio (OR): 1.18; 95% confidence interval (CI): 1.08–1.30; *P*<0.001), a non-*albicans* candidemia caused by *C. tropicalis* (OR: 1.91; 95% CI: 1.11–3.29; *P*=0.019), neutropenia (OR: 3.67; 95% CI: 1.07–12.55; *P*=0.038) and septic shock (OR: 2.29; 95% CI: 1.39–3.77; *P*=0.001) were independent predictors of 28-day mortality.

## DISCUSSION

Candidemia, with a shift in species distribution towards non-*albicans Candida* species, remains a lethal disease. Moreover, malignancy is identified as the major comorbidity of patients with candidemia. Here, we report several relevant findings obtained from a large multicenter study that characterized non-*albicans* candidemia in cancer patients. In this study, *C. tropicalis* was the main cause of non-*albicans* candidemia in cancer patients. Most isolates of non-*albicans Candida* (>80%) were susceptible or susceptible dose-dependent to echinocandins and fluconazole. Non-*albicans* candidemia due to *C. tropicalis*, as well as a higher Charlson comorbidity index, neutropenia and septic shock, were independently associated with poor prognosis.

*C. parapsilosis* has been reported to be the most common cause of non-*albicans* candidemia among patients with malignancy in Western countries, including the United States, Australia and Portugal.^13, 14, 23^ In our study, the major non-*albicans Candida* species identified in cancer patients with candidemia was *C. tropicalis*, consistent with previous reports in Taiwan.^6, 15^ The clinical features of the non-*albicans Candida* species distribution of candidemia seemed more likely to be influenced by geography than by underlying comorbidities. Consequently, establishing the epidemiology at each research surveillance site is important, and *C. tropicalis* is one of the most important and predominant causes of non-*albicans* candidemia in cancer patients, especially in Taiwan. In fact, *C. tropicalis* has been identified as the leading cause of non-*albicans* candidemia in all patients with or without a diagnosed malignancy (49.4%–35.4%) in Taiwan since the year 2000,^7, 20, 24, 25, 26^ except in a study by Lin *et al.*, which enrolled a small number of patients with non-*albicans* candidemia.^27^

In our study, *C. tropicalis* was not only the most common cause of non-*albicans* candidemia but also the species with the highest resistance rate to fluconazole among the preserved isolates of the three non-*albicans Candida* species (*C. tropicalis*, *C. glabrata* and *C. parapsilosis*) analyzed. In general, *C. glabrata*, with reduced susceptibility,^28^ and *C. krusei*, with intrinsic resistance,^29^ were two commonly indicated non-*albicans Candida* species for which increasing fluconazole resistance was observed. According to the definitions set by the Clinical Laboratory Standards Institute [M27-S4],^22^ national surveillance research in Brazil showed that none of the *C. tropicalis* isolates obtained were resistant to fluconazole, whereas 36.4% (4/11) of *C. glabrata* isolates were resistant to fluconazole. However, 96.7% (93/96) of *C. glabrata* isolates were susceptible dose-dependent to fluconazole in our study, and 86.1% (105/122) of *C. tropicalis* isolates were susceptible or susceptible dose-dependent to fluconazole. Tan *et al.*^30^ conducted a multicenter study that characterized clinical isolates from patients with candidemia in countries in the Asia-Pacific region and reported the fluconazole susceptibility rates for *C. tropicalis* (susceptible rate=75.8% susceptible dose-dependent rate=6.1%) and *C. glabrata* (susceptible dose-dependent rate=94.9%), which are in accordance with the results of our study. Furthermore, Yang *et al.*^5^ reported that of the *C. glabrata* isolates obtained from patients with candidemia, none were resistant to fluconazole, but 27.8% (5/18) of *C. tropicalis* isolates were resistant to fluconazole in China. A more recent study by Chen *et al.*^31^ reported that the resistance rate to fluconazole of *C. tropicalis* was 9.5% in patients with candidemia in Taiwan and that the resistance rates of *C. glabrata* and *C. parapsilosis* were 5.4% and 2%, respectively. Taken together, these studies imply that not only the species distribution of non-*albicans* candidemia but also the antifungal susceptibility of the causative agents differs from region to region and that *C. tropicalis* is the most fluconazole-resistant species among clinically common non-*albicans Candida* species causing candidemia around the Asia-Pacific region, including Taiwan.

Previous studies have reported that underlying conditions and the severity of illness, including advanced age, septic shock, neutropenia, acute respiratory failure and a high Acute Physiology and Chronic Health Evaluation (APACHE) II score, are significantly associated with mortality in cancer patients with non*-albicans* candidemia,^6, 13, 15^ consistent with the results presented in this study. We further found that non-*albicans* candidemia due to *C. tropicalis* was an independent predictor of 28-day mortality. Compared with other species of non-*albicans Candida*, patients with *C. tropicalis* candidemia tended to present a higher severity of illness, such as neutropenia (10.3% versus 3%, *P*=0.006) and septic shock (44.8% versus 29.4%, *P*=0.003). These results and the relatively high resistance rate to fluconazole of *C. tropicalis*, as mentioned previously, may contribute to the poor outcome of patients with non-*albicans* candidemia due to *C. tropicalis.* In the literature, *C.* *tropicalis* is shown as the highest fluconazole-resistant species among the three most clinically common non-*albicans Candida* (*C. tropicalis*, *C. glabrata* and *C. parapsilosis*) in Asian countries,^5, 30, 31^ but these studies did not investigate the impact of different non-*albicans Candida* species on survival in cancer patients. A population-based study in Spain conducted by Puig-Asensio *et al.*^16^ demonstrated that candidemia caused by *C. krusei* was an independent risk factor of higher 30-day mortality in cancer patients with the use of a central venous catheter (OR: 12.59; 95% CI: 2.46–64.48; *P*=0.002). This disagrees with results from our study, possibly due to different geographies and study populations. Further large-scale, intensive research on this issue in different regions is imperatively needed.

A few limitations of the present study should be noted. First, this study was inherently retrospective in design, and thus missing values and potential information bias may have arisen. Second, APACHE II scores from the enrolled patients were not available. Septic shock and neutropenia are possible confounding factors in APACHE II scores, and therefore the frequencies of these two variables were monitored in the study to strengthen the results. Third, information about catheter removal in patients with CRBSI was unavailable, and the influence of this action on survival could not be evaluated. Finally, *in vitro* susceptibility results were only available for the preserved isolates, and appropriate or inappropriate therapy could not be defined for all of the study population. As a result, the analysis of mortality did not include the use of antifungal agents. Further research is needed to investigate the impact of antifungal therapy and catheter removal on the prognosis of patients with non-*albicans* candidemia. Despite its limitations, this is a large, multicenter study that may allow for more generalizable findings and may provide useful information for the treatment of fungal infections in cancer patients.

In conclusion, the epidemiology of non-*albicans* candidemia in cancer patients is a growing concern that must be addressed. Among cancer patients in Taiwan, *C. tropicalis* is the leading species causing non-*albicans* candidemia and is the species with the highest resistance to fluconazole among the clinically major non-*albicans Candida* species. Continuous monitoring of the species distribution and antifungal susceptibility of candidemia cases is necessary.

## Figures and Tables

**Table 1:** Demographics and clinical characteristics of cancer patients with non-*albicans* candidemia

**Characteristic**	**Number of patients (%)**
Gender, male	221 (63.9)
	
*Age, years*	63.9±15.4
Age ≥65 years	165 (47.7)
	
Charlson comorbidity index	5.6±2.8
	
*Underlying comorbidities*	
Diabetes mellitus	107 (30.9)
Cerebrovascular accident	24 (6.9)
Congestive heart failure	18 (5.2)
End-stage renal disease	14 (4)
Liver cirrhosis	26 (7.5)
Chronic lung disease	36 (10.4)
	
*Type of cancer*	
Head and neck cancer	58 (16.8)
Lung cancer	22 (6.4)
Gastrointestinal cancer	160 (46.2)
Genitourinary cancer	40 (11.6)
Breast cancer	15 (4.3)
Hematological malignancy	46 (13.3)
Others^a^	7 (2)
	
*Non-albicans Candida species*	
*C. tropicalis*	145 (41.9)
*C. glabrata*	111 (32.1)
*C. parapsilosis*	70 (20.2)
*C. krusei*	7 (2)
*C. guilliermondii*	5 (1.4)
*C. sake*	2 (0.6)
*C. dubliniensis*	2 (0.6)
*C. lipolytica*	1 (0.3)
*C. lusitaniae*	1 (0.3)
*C. norvegensis*	1 (0.3)
*C. famata*	1 (0.3)
	
*Origin of non-albicans candidemia*	
Primary infection	136 (39.3)
Catheter-related infection	108 (31.2)
Urinary tract infection	29 (8.4)
Respiratory tract infection	42 (12.1)
Intra-abdominal infection	29 (8.4)
Skin and soft tissue infection	2 (0.6)
	
*Underlying conditions*	
Presence of central venous catheter	294 (85)
Presence of Foley catheter	127 (36.7)
Chemotherapy	168 (48.6)
Total parental nutrition	96 (27.7)
Steroid	119 (34.4)
Proton-pump inhibitor	145 (41.9)
Abdominal surgery	58 (16.8)
Prior exposure to broad-spectrum antibiotics	306 (88.4)
	
Neutropenia	21 (6.1)
Septic shock	124 (35.8)
Concomitant bacterial infections	127 (36.7)
	
*Antifungal therapy*	
Amphotericin B	5 (1.4)
Triazole	192 (55.5)
Echinocandin	102 (29.5)
None	47 (13.6)
	
Length of hospital stay after infection (days)	22.1±20.4
28-day mortality	154 (44.5)

Data are presented as mean±standard deviation (s.d.) or frequency with percentage (%).

a Others included glioblastoma multiforme, peritoneal mesothelioma, retroperitoneal carcinoma, skin cancer and malignancy of unknown origin.

**Table 2:** Distribution and frequency of non-*albicans Candida* species in patients with different types of cancers

**Species**	**Number of isolates (%)**				
	**Head and neck cancer**	**Gastrointestinal tract cancer**	**Lung cancer**	**Genitourinary cancer**	**Hematological cancer**
*C. tropicalis*	26 (44.8)	62 (38.8)	10 (45.5)	16 (40)	27 (58.7)
*C. glabrata*	14 (24.1)	60 (37.5)	6 (27.3)	14 (35)	10 (21.7)
*C. parapsilosis*	17 (29.3)	29 (18.1)	6 (27.3)	8 (20)	4 (8.7)
*C. krusei*	0 (0)	3 (1.9)	0 (0)	0 (0)	3 (6.5)
*C. guilliermondii*	0 (0)	3 (1.9)	0 (0)	2 (5)	0 (0)
Others^a^	1 (1.7)	3 (1.9)	0 (0)	0 (0)	2 (4.3)

Data are presented as frequency with percentage (%).

a Other species included *C. sake*, *C. lusitaniae*, *C. lipolytica*, *C. norvegensis*, *C. famata* and *C. dubliniensis*.

**Table 3:** *In vitro* activities of antifungal agents tested against 251 non-*albicans Candida* isolates obtained from patients with candidemia according to the Clinical and Laboratory Standards Institute recommendations

**Non-*****albicans Candida*** **species**	**MIC** **range (mg/L)**	**MIC**_**50**_**(mg/L)**	**MIC**_**90**_ **(mg/L)**	**Number of isolates (%)**			
				**S**	**SDD**	**I**	**R**
*C. tropicalis (n=122)*							
Amphotericin B	0.25–2	0.5	1	—	—	—	—
Fluconazole	0.25–128	2	8	87 (71.3)	18 (14.8)	—	17 (13.9)
Voriconazole	0.015–4	0.25	1	60 (49.2)	49 (40.2)	—	13 (10.7)
Posaconazole	0.015–2	0.25	0.5	—	—	—	—
Itraconazole	0.06–1	0.25	0.5	—	—	—	—
Caspofungin	0.015–>8	0.06	0.25	118 (96.7)	—	1 (0.8)	3 (2.5)
Micafungin	0.015–2	0.03	0.03	118 (96.7)	—	1 (0.8)	3 (2.5)
Anidulafungin	0.03–1	0.12	0.25	119 (97.5)	—	1 (0.8)	2 (1.6)
Flucytosine	≤0.06–>64	≤0.06	0.12	—	—	—	—
							
*C. glabrata (n=96)*							
Amphotericin B	≤0.12–4	0.5	1	—	—	—	—
Fluconazole	0.5–>256	16	32	—	93 (96.7)	—	3 (3.1)
Voriconazole	≤0.008–4	0.5	1	—	—	—	—
Posaconazole	0.015–>8	1	2	—	—	—	—
Itraconazole	0.03–2	0.5	1	—	—	—	—
Caspofungin	0.03–1	0.12	0.25	80 (83.3)	—	14 (14.6)	2 (2.1)
Micafungin	≤0.008–1	0.015	0.015	90 (93.8)	—	1 (1)	5 (5.2)
Anidulafungin	≤0.015–1	0.06	0.12	90 (93.8)	—	1 (1)	5 (5.2)
Flucytosine	≤0.06–0.5	≤0.06	0.12	—	—	—	—
							
*C. parapsilosis (n=33)*							
Amphotericin B	0.25–1	0.5	0.5	—	—	—	—
Fluconazole	0.25–8	1	2	30 (90.9)	1 (3)	—	2 (6.1)
Voriconazole	≤0.008–0.25	0.015	0.03	32 (97)	1 (3)	—	0 (0)
Posaconazole	0.015–0.12	0.03	0.06	—	—	—	—
Itraconazole	0.03–0.25	0.06	0.12	—	—	—	—
Caspofungin	0.25–1	0.5	0.5	33 (100)	—	0 (0)	0 (0)
Micafungin	0.25–2	1	2	33 (100)	—	0 (0)	0 (0)
Anidulafungin	0.25–2	1	2	33 (100)	—	0 (0)	0 (0)
Flucytosine	≤0.06–0.5	0.12	0.25	—	—	—	—

Abbreviations: intermediate, I; minimum inhibitory concentrations for 50% and 90% of the organisms, respectively, MIC_50/90_; susceptible, S; susceptible dose-dependent, SDD; resistant, R.

Data are presented as frequency with percentage (%).

**Table 4:** Analysis of potential risk factors associated with 28-day mortality in patients with non-*albicans* candidemia

**Variables**	**Survival** ***n*****=192** **Number (%)**	**Death** ***n*****=154** **Number (%)**	**Univariate analysis**	**Multivariate analysis**	
			***P-*****value**	**Odds ratio (95% CI)**	***P***-**value**
Male	122 (63.5)	99 (64.3)	0.886	0.79 (0.48–1.31)	0.361
Age in years	63.8±15.3	64±15.5	0.923	0.99 (0.98–1.01)	0.381
Charlson comorbidity index	5.2±2.6	6±3	0.011	1.18 (1.08–1.30)	<0.001
Hematological malignancy	20 (10.4)	26 (16.9)	0.081	0.80 (0.35–1.82)	0.598
					
*Non-albicans Candida species*					
*C. tropicalis*	67 (34.9)	78 (50.6)	0.003	1.91 (1.11–3.29)	0.019
*C. glabrata*	66 (34.4)	45 (29.2)	0.308		
*C. parapsilosis*	48 (25)	22 (14.3)	0.015	0.94 (0.47–1.86)	0.858
*C. krusei*	4 (2.1)	3 (2)	0.931		
*C. guilliermondii*	3 (1.6)	2 (1.3)	0.839		
*C. sake*	2 (1)	0 (0)	0.999		
*C. dubliniensis*	0 (0)	2 (1.3)	0.999		
*C. lipolytica*	0 (0)	1 (0.7)	1.000		
*C. norvegensis*	0 (0)	1 (0.7)	1.000		
*C. famata*	1 (0.5)	0 (0)	1.000		
					
*Origin of non-albicans candidemia*					
Primary infection	63 (32.8)	73 (47.4)	0.006	1.37 (0.77–2.42)	0.283
Catheter-related infection	76 (39.6)	32 (20.8)	<0.001	0.58 (0.31–1.07)	0.082
Urinary tract infection	15 (7.8)	14 (9.1)	0.670		
Respiratory tract infection	23 (12)	19 (12.3)	0.919		
Intra-abdominal infection	14 (7.3)	15 (9.7)	0.416		
Skin and soft tissue infection	1 (0.5)	1 (0.6)	0.876		
					
*Underlying conditions*					
Presence of central venous catheter	167 (87)	127 (82.5)	0.245		
Presence of Foley catheter	66 (34.4)	61 (39.6)	0.316		
Chemotherapy	94 (49)	74 (48.1)	0.867		
Total parental nutrition	58 (30.2)	38 (24.7)	0.254		
Steroid	58 (30.2)	61 (39.6)	0.068	1.61 (0.97–2.67)	0.068
Proton-pump inhibitor	78 (40.6)	67 (43.5)	0.589		
Abdominal surgery	35 (18.2)	23 (14.9)	0.503		
Prior exposure to broad-spectrum antibiotics	169 (88)	137 (89)	0.786		
Neutropenia	5 (2.6)	16 (10.4)	0.005	3.67 (1.07–12.55)	0.038
Septic shock	50 (26)	74 (48.1)	<0.001	2.29 (1.39–3.77)	0.001
Concomitant bacterial infections	65 (33.9)	62 (40.3)	0.220		

Data are presented as mean±standard deviation (s.d.) or frequency with percentage (%).

## References

[bib1] Marchetti O, Bille J, Fluckiger U et al. Epidemiology of candidemia in Swiss tertiary care hospitals: secular trends, 1991–2000. Clin Infect Dis 2004; 38: 311–320. 14727199 10.1086/380637

[bib2] Wisplinghoff H, Bischoff T, Tallent SM et al. Nosocomial bloodstream infections in US hospitals: analysis of 24,179 cases from a prospective nationwide surveillance study. Clin Infect Dis 2004; 39: 309–317. 15306996 10.1086/421946

[bib3] Magill SS, Edwards JR, Bamberg W et al. Multistate point-prevalence survey of health care-associated infections. N Engl J Med 2014; 370: 1198–1208. 24670166 10.1056/NEJMoa1306801 PMC4648343

[bib4] Hsueh PR, Teng LJ, Yang PC et al. Emergence of nosocomial candidemia at a teaching hospital in Taiwan from 1981 to 2000: increased susceptibility of *Candida* species to fluconazole. Microb Drug Resist 2002; 8: 311–319. 12523628 10.1089/10766290260469570

[bib5] Yang ZT, Wu L, Liu XY et al. Epidemiology, species distribution and outcome of nosocomial *Candida* spp. bloodstream infection in Shanghai. BMC Infect Dis 2014; 14: 241. 24886130 10.1186/1471-2334-14-241 PMC4033490

[bib6] Tang HJ, Liu WL, Lin HL et al. Epidemiology and prognostic factors of candidemia in cancer patients. PLoS One 2014; 9: e99103. 24901336 10.1371/journal.pone.0099103 PMC4047064

[bib7] Hii IM, Chang HL, Lin LC et al. Changing epidemiology of candidemia in a medical center in middle Taiwan. J Microbiol Immunol Infect 2015; 48: 306–315. 24113067 10.1016/j.jmii.2013.08.017

[bib8] Cleveland AA, Harrison LH, Farley MM et al. Declining incidence of candidemia and the shifting epidemiology of *Candida* resistance in two US metropolitan areas, 2008-2013: results from population-based surveillance. PLoS One 2015; 10: e0120452. 25822249 10.1371/journal.pone.0120452 PMC4378850

[bib9] Khatib R, Johnson LB, Fakih MG et al. Current trends in candidemia and species distribution among adults: *Candida glabrata* surpasses *C. albicans* in diabetic patients and abdominal sources. Mycoses 2016; 59: 781–786. 10.1111/myc.12531 27402377

[bib10] Tortorano AM, Kibbler C, Peman J et al. Candidaemia in Europe: epidemiology and resistance. Int J Antimicrob Agents 2006; 27: 359–366. 16647248 10.1016/j.ijantimicag.2006.01.002

[bib11] Wey SB, Mori M, Pfaller MA et al. Hospital-acquired candidemia. The attributable mortality and excess length of stay. Arch Intern Med 1988; 148: 2642–2645. 3196127 10.1001/archinte.148.12.2642

[bib12] Zaoutis TE, Argon J, Chu J et al. The epidemiology and attributable outcomes of candidemia in adults and children hospitalized in the United States: a propensity analysis. Clin Infect Dis 2005; 41: 1232–1239. 16206095 10.1086/496922

[bib13] Sipsas NV, Lewis RE, Tarrand J et al. Candidemia in patients with hematologic malignancies in the era of new antifungal agents (2001-2007): stable incidence but changing epidemiology of a still frequently lethal infection. Cancer 2009; 115: 4745–4752. 19634156 10.1002/cncr.24507

[bib14] Slavin MA, Sorrell TC, Marriott D et al. Candidaemia in adult cancer patients: risks for fluconazole-resistant isolates and death. J Antimicrob Chemother 2010; 65: 1042–1051. 20202987 10.1093/jac/dkq053

[bib15] Chen CY, Huang SY, Tsay W et al. Clinical characteristics of candidaemia in adults with haematological malignancy, and antimicrobial susceptibilities of the isolates at a medical centre in Taiwan, 2001–2010. Int J Antimicrob Agents 2012; 40: 533–538. 23006521 10.1016/j.ijantimicag.2012.07.022

[bib16] Puig-Asensio M, Ruiz-Camps I, Fernandez-Ruiz M et al. Epidemiology and outcome of candidaemia in patients with oncological and haematological malignancies: results from a population-based surveillance in Spain. Clin Microbiol Infect 2015; 21: 491.e1–10. 25703212 10.1016/j.cmi.2014.12.027

[bib17] Bassetti M, Righi E, Costa A et al. Epidemiological trends in nosocomial candidemia in intensive care. BMC Infect Dis 2006; 6: 21. 16472387 10.1186/1471-2334-6-21 PMC1379648

[bib18] Hachem R, Hanna H, Kontoyiannis D et al. The changing epidemiology of invasive candidiasis: *Candida glabrata* and *Candida krusei*as the leading causes of candidemia in hematologic malignancy. Cancer 2008; 112: 2493–2499. 18412153 10.1002/cncr.23466

[bib19] Horn DL, Neofytos D, Anaissie EJ et al. Epidemiology and outcomes of candidemia in 2019 patients: data from the prospective antifungal therapy alliance registry. Clin Infect Dis 2009; 48: 1695–1703. 19441981 10.1086/599039

[bib20] Chen LY, Kuo SC, Wu HS et al. Associated clinical characteristics of patients with candidemia among different *Candida* species. J Microbiol Immunol Infect 2013; 46: 463–468. 23010536 10.1016/j.jmii.2012.08.001

[bib21] Horan TC, Andrus M, Dudeck MA. CDC/NHSN surveillance definition of health care-associated infection and criteria for specific types of infections in the acute care setting. Am J Infect Control 2008; 36: 309–332. 18538699 10.1016/j.ajic.2008.03.002

[bib22] Clinical and Laboratory Standards Institute (CLSI)Reference Method for Broth Dilution Antifungal Susceptibility Testing of Yeasts. (Approved Standard M27-S4), 4th edn. Wayne, PA, USA: National Committee for Clinical and Laboratory Standards. 2012.

[bib23] Sabino R, Verissimo C, Brandao J et al. Epidemiology of candidemia in oncology patients: a 6-year survey in a Portuguese central hospital. Med Mycol 2010; 48: 346–354. 19657956 10.1080/13693780903161216

[bib24] Chen PY, Chuang YC, Wang JT et al. Comparison of epidemiology and treatment outcome of patients with candidemia at a teaching hospital in Northern Taiwan, in 2002 and 2010. J Microbiol Immunol Infect 2014; 47: 95–103. 23063082 10.1016/j.jmii.2012.08.025

[bib25] Tsai CC, Wang CC, Kuo HY et al. Adult candidemia at a medical center in northern Taiwan: a retrospective study. J Microbiol Immunol Infect 2008; 41: 414–421. 19122924

[bib26] Chen LY, Liao SY, Kuo SC et al. Changes in the incidence of candidaemia during 2000–2008 in a tertiary medical centre in northern Taiwan. J Hosp Infect 2011; 78: 50–53. 21316800 10.1016/j.jhin.2010.12.007

[bib27] Chi HW, Yang YS, Shang ST et al. *Candida albicans* versus non-*albicans* bloodstream infections: the comparison of risk factors and outcome. J Microbiol Immunol Infect 2011; 44: 369–375. 21524971 10.1016/j.jmii.2010.08.010

[bib28] Ruan SY, Chu CC, Hsueh PR. *In vitro* susceptibilities of invasive isolates of *Candida* species: rapid increase in rates of fluconazole susceptible-dose dependent *Candida glabrata* isolates. Antimicrob Agents Chemother 2008; 52: 2919–2922. 18458136 10.1128/AAC.00323-08 PMC2493111

[bib29] Pfaller MA, Diekema DJ, Gibbs DL et al. Results from the ARTEMIS DISK Global Antifungal Surveillance study, 1997 to 2005: an 8.5-year analysis of susceptibilities of *Candida* species and other yeast species to fluconazole and voriconazole determined by CLSI standardized disk diffusion testing. J Clin Microbiol 2007; 45: 1735–1745. 17442797 10.1128/JCM.00409-07 PMC1933070

[bib30] Tan TY, Hsu LY, Alejandria MM et al. Antifungal susceptibility of invasive *Candida* bloodstream isolates from the Asia-Pacific region. Med Mycol 2016; 54: 471–477. 26868904 10.1093/mmy/myv114

[bib31] Chen YC, Kuo SF, Chen FJ et al. Antifungal susceptibility of *Candida* species isolated from patients with candidemia in southern Taiwan, 2007–2012: impact of new antifungal breakpoints. Mycoses 2017; 60: 89–95. 27621210 10.1111/myc.12553

